# Single-nucleotide polymorphisms in chromosome 3p14.1- 3p14.2 are associated with susceptibility of Type 2 diabetes with cataract

**Published:** 2010-07-01

**Authors:** Hui-Ju Lin, Yu-Chuen Huang, Jane-Ming Lin, Jer-Yuarn Wu, Liuh-An Chen, Chao-Jen Lin, Yung-Ping Tsui, Chih-Ping Chen, Fuu-Jen Tsai

**Affiliations:** 1 Department of Medical Genetics, China Medical University Hospital, Taichung, Taiwan; 2 School of Chinese Medicine, College of Chinese Medicine, China Medical University, Taichung, Taiwan; 3 Department of Ophthalmology, China Medical University Hospital, Taichung, Taiwan; 4 National Genotyping Center, Academia Sinica, Taipei, Taiwan; 5 Department of Pediatrics, Changhua Christian Hospital, Taiwan; 6 Departments of Obstetrics and Gynecology and Medical Research, Mackay Memorial Hospital, Taipei, Taiwan

## Abstract

**Purpose:**

Type 2 diabetes (T2D) is highly prevalent worldwide and cataracts are of high incidence in T2D patients. In this study, we identify genetic variants that predispose type 2 diabetes (T2D) patients to cataracts in the Han-Chinese residing in Taiwan.

**Methods:**

We conducted a genome-wide association study with a total of 1,715 cases and 2,000 random controls. In the haplotype study, we defined haplotype 1 (Ht 1) to haplotype 4 (Ht 4) as the alternative alleles of the DM and cataract related chromosome 3p14.1- 3p14.2 polymorphisms.

**Results:**

The most significant association was detected with rs11129182, rs17047573, and rs17047586 in chromosome 3p14.1- 3p14.2 (p value=3.52×10^−7^, 8.35×10^−8^, and 7.65×10^−8^, respectively). In genotype analysis, the “CT” genotype of rs11129182, the ‘GG’ genotype of rs17047573, and the ‘GG’ genotype of rs17047586 were significantly different in the T2D and cataract groups (OR=3.03, 7.47, and 7.51, individually; 95% confidence index (CI): 1.97–4.65, 3.36–16.6, and 3.38–16.7, individually). In the haplotype study, the distribution of the Ht3 and Ht4 between the DM and cataract group and the control group differed significantly between the two groups (p=0.0004). The odds ratio (OR) of Ht4 was 1.89 and the 95% confidence interval (CI) was 1.36–2.65.

**Conclusions:**

The major functions of the genes are voltage-dependent anion-selective channel proteins, long myosin light chain kinase, adenylyl cyclase-associated proteins and retinoic acid receptors and are all closely related with the pathogenesis of T2D and cataractogenesis. This has helped us understand the pathogenesis of T2D patients with cataracts.

## Introduction

Diabetes mellitus (DM) is one of the major causes of sight loss. Cataracts, considered a complication of DM, are a cause of visual impairment that can affect individuals at all ages [1,2]. Among the various complications of diabetes mellitus in the eyes, cataracts are only less critical then diabetic retinopathy as cause of blindness. Although chronic hyperglycemia and the duration of DM are considered to be the major risk factors for this diabetic complication [3,4], there are some patients with severe cataracts while others present with few risk factors.

A cataract is a clouding that develops in the crystalline lens of the eye or in its envelope; varying in degree from slight to complete opacity and obstructing the passage of light. Age-related cataracts are responsible for 48% of world blindness, which represents about 18 million people, according to the World Health Organization (WHO) [5]. In many countries, surgical services are inadequate and cataracts remain the leading cause of blindness. Cataracts are a clinically and genetically heterogeneous group of eye disorders that causes visual impairment. At least 34 loci and mutations in 22 genes have been reported to be linked with different forms of cataracts. Type 2 diabetes (T2D) affects at least 6% of the world’s population, and the prevalence is expected to double worldwide by the year 2025 [6]. T2D is a complex disease characterized by hyperglycemia that results from impaired pancreatic β-cell function, decreased insulin action at target tissues, and increased glucose output by the liver [7]. Both genetic components and environmental factors contribute to the pathogenesis of T2D. This disease is considered to be a polygenic disorder that each genetic variance confers a partial and additive effect. Only 5%–10% of T2D are monogenic diabetes with single gene defects. A large amount of effort has been devoted to finding common T2D genes, including genome-wide linkage, candidate-gene and genome-wide association studies [8-10]. However, these genes cannot be held solely responsible for the development of T2D includes the wide variability of the prevalence of T2D in different ethnic groups. Cataracts are more common in people with diabetes than in the general population under the age of 40 years and they are morphologically similar to senile cataracts. The exact correlation between cataracts and T2D is without a definite conclusion. Cataracts are a significant complication of T2D [8-10]. In this study, we aimed to uncover diabetes susceptibility loci that increase the risk for T2D, as well as cataracts, in the Chinese population residing in Taiwan.

## Methods

The levels of lens opacity were classified according to the Lens Opacities Classification System (LOCS) [11,12] in this study. LOCS involves comparing the slit lamp view of the lens to a color plate of LOCS III standards (Table 1). LOCS uses standard reference photographs taken during the slit lamp examination. LOCS defines the extent of changes in opacity in the cortical (C) and posterior subcapsular (P) regions, shown in Table 2. It also notes the extent of color changes of the nucleus, as well as the intensity of nuclear opalescence. In this study, scores were given to the extent of each lens’ opacity according to their LOCS classification; for example, N0 was scored as 0 and NIV was given a 4. After summing up the scores of the four parts (nuclear color, nuclear opacity, cortical cataract, and posterior subcapsule), the total score was obtained ranging from 0 to 15. The patients in the study group were patients with cataract score of 10 or more. The patients in the control group were subjects with cataract score equal to or less than 3. The patients the in control group, T2D patients with cataract scores equal or less then score 3 in LOCS cataract evaluation system, were those with corrected visual acuity (BCVA) of 0.18 logMAR (6/9) or better. The patients in study group, T2D patients with a cataract score of 10 or more in LOCS cataract evaluation system, had BCVA of 0.48 logMAR (6/18) or worse.

**Table 1:** Lens opacities classification (LOC) system defines the extent of opacification of lens.

**Parameter**	**Features**	**Grade**
Nuclear color (NC)	The color of the posterior cortical/posterior subcapsular reflex is:	** **
** **	<NI standard	NC0
** **	Similar to the NI standard	NC1
** **	>NI standard	NC2
Nuclear opalescence (N)	The average opalescence of the nucleus is:	** **
** **	<=that in N0	N0
** **	>N0 but <=NI	NI
** **	>NI but <=NII	NII
** **	>NII but <=NIII	NIII
** **	>NIII	NIV
Cortical cataract (C)	The aggregate extent of linearly organized or clustered opacities is:	** **
** **	<=that in C0	C0
** **	>C0 but <=Ctr (trace)	Ctr
** **	>Ctr but <=CI	CI
** **	>Ctr but <=CII	CII
** **	>Ctr but <=CIII	CIII
** **	>Ctr but <=CIV	CIV
** **	>CIV	CV
Posterior subcapsular (P)	The aggregate extent of the axial opacity seen in retro-illumination is:	
	<=that in P0	P0
	>P0 but <=PI	PI
	>PI but <=PII	PII
	>PII but <=PIII	PIII
	>PIII	PIV

**Table 2:** Lens opacities classification system defines the extent of opacification of lens cortex opacification and posterior subcapsule of lens.

**Cortical standard**	**Description**
C0	Clear lens devoid of aggregated dots, flecks,
Ctr	Minimal degree of cortical opacification and/or
CI	More extensive opacification with small
CII	Cortical spoking that obscures more than 2 full
CIII	Opacification that obscures about 50% of the
CIV	Advanced opacification filling about 90% of the
**Posterior subcapsule standard**	**Description**
P0	Clear posterior capsule
PI	Cataract filling about 3% of the area of the posterior capsule
PII	About 30% opacification of the area of the posterior capsule
PIII	About 50% opacification of the area of the posterior capsule

### Subject participants

All T2D cases were diagnosed using the American Diabetic Association Criteria. Subjects with type 1 diabetes, gestational diabetes, and MODY (Maturity-onset diabetes of the young) were excluded from this study. In the first step to study T2D related genes, 2,000 Chinese subjects were randomly selected from the Taiwan Han-Chinese Cell and Genome Bank as controls [13]. The random controls and all participating T2D cases were of Han Chinese origin as the Han Chinese account for 98% of the Taiwan population. Our study was reviewed by the ethics committee of CMUH, and informed consent was obtained from all patients. A comprehensive ophthalmic examination and blood collection were performed. The study was performed according to the tenets of the Declaration of Helsinki for research involving human subjects. In addition, we investigated HbA1c, total cholesterol, triglyceride, HDL-cholesterol, LDL-cholesterol, blood urea nitrogen (BUN), and creatinine. Each patient underwent best-corrected distance visual acuity measurement using a Snellen chart. Visual acuity was defined as the average value of the corrected visual acuity of both eyes before examination. An ophthalmic examination was performed with slit lamp biomicroscopy and indirect ophthalmoscope fundus examination after the pupil was maximally dilated with 1.0% tropicamide (Mydriacyl).

### Genotyping

Genomic DNA was extracted from peripheral blood using PUREGENE DNA isolation kit (Gentra Systems, Minneapolis, MN). In the first stage, whole genome genotyping using Illumina HumanHap550-Duo BeadChip was performed by deCODE genetics, Inc., Reykjavík, Iceland. Genotype calling was done using the standard procedure implemented in Beadstudio, with default parameters suggested by the platform manufacturer. All sample included had a call rate of >97% with an average of 99.67±0.37%. Single-nucleotide polymorphisms (SNPs) were excluded if they showed either: (i) a total call rate of <95% in the cases and controls combined; (ii) a minor allele frequency of <5% and a total call rate of <99% in the cases and controls combined; (iii) significant distortion from Hardy–Weinberg equilibrium in the controls (p value <10^−7^). In sum, 517,401 SNPs (92.36%) passed the quality control filter with an average call rate of 99.91%. Genotyping validation was performed using the Sequenom iPLEX assay (SEQUENOM MassARRAY system; Sequenom, San Diego, CA) [14-16]. Visualized and analyzed data could be generated by the Illumina's platforms.

### Statistical analysis

Association analysis was performed to compare allele frequency and genotype distribution between cases and controls using five single-point methods: genotype, allele-type, and Cochran-Armitage trend test along with tests considering dominant and recessive inheritance modes. Empirical p-values were also obtained with 10^7^ simulations. SNPs with p-values between 10^−7^ and 10^−5^, a cut-off for the multiple-comparison adjusted by Bonferroni correction, were considered to be significantly associated with the traits. All five SNPs were analyzed with the HAPLOVIEW program, v4.1.

## Results

A total of 968 unrelated individuals with T2D, over 20 years of age, were recruited from China Medical University Hospital (CMUH), Taiwan. The T2D patients with a LOCS cataract score equal to or less than 3 and with a BCVA (best corrected visual acuity) of 0.18 logMAR (6/9) or better were included in the control group. The T2D patients with a cataract score of 10 or more in LOCS cataract evaluation system and with a BCVA of 0.48 logMAR (6/18) or worse were included in the cataract group. The patients that met the criteria were 109 and 649, respectively. Average intraocular pressure (IOP) was 15.36 mmHg and 15.86 mmHg, respectively. The characteristics of the T2D patients with cataracts and without cataracts can be seen in Table 3. The physical conditions of the T2D patients are expressed in Table 4. We conducted a genome-wide association study (GWAS) to simultaneously identify genetic variants for T2D patients with cataracts in Han-Chinese residing in Taiwan. We started with 517,401 SNPs that passed quality control filters using the Illumina Hap550duov3 chip. In the study, we found three SNPs with significant evidence (p<10^−7^) for association with diabetic cataracts, locus at chromosome 3p14.1- 3p14.2 (rs11129182, rs17047573, and rs17047586) (p value=3.52×10^−7^, 8.35×10^−8^, and 7.65×10^−8^, respectively; Table 4). In the three SNPs, the CT genotype of rs11129182, GG genotype of rs17047573, and the GG genotype of rs17047586 were significantly different in the T2D with cataracts and T2D without cataracts groups (OR=3.03, 7.47, and 7.51, respectively; 95% confidence interval (CI): 1.97–4.65, 3.36–16.6, and 3.38–16.7, respectively; Table 5). We found that there were four haplotypes composed by the polymorphisms (Table 6). We defined haplotype 1 (Ht 1) to haplotype 4 (Ht 4) as the alternative alleles of the DM and cataract related 3p14.1- 3p14.2 polymorphisms (Table 5). Ht1 (G/C) and Ht2 (A/T) were composed by rs9876471 and rs264667 and Ht3 (T/A) and Ht4 (G/G) were composed by rs17047573 and rs17047586. The distribution of the Ht3 and Ht4 between the DM and cataract group and the control group differed significantly between the two groups (p=0.0004). The odds ratio (OR) of Ht4 was 1.89 and the 95% confidence interval (CI) was 1.36–2.65.

**Table 3:** Characteristics of T2D patients with and without cataract.

**Characteristics**	**T2D with cataract**	**T2D without cataract**
Age (y/o)	62±2.35 (20–70)	63±1.75 (20–70)
F/M	57/52	332/317
BCVA	1.53±0.13 (0.48–2)	0.04±0.03 (0–0.18)
IOP (mmHg)	12.5±0.3 (7–21)	13.2±0.2 (7–21)

In the table, the term “F/M” indicates female/male and “BCVA” indicates best corrected visual acuity.

**Table 4:** The physical condition of the T2D patients.

**Variable**	**Mean C/N**	**Std Dev C/N**	**Min C/N**	**Max C/N**
BUN	17.42/17.32	8.17/8.15	5.80/5.70	87.10/82.20
Creatinine	0.91/0.90	0.58/0.62	0.23/0.29	13.80/12.95
total-cholesterol	189.02/187.10	41.23/41.08	66.00/68.20	560.00/550.20
triglyceride	163.82/163.84	125.63/126.58	58.90/21.00	1380.00/1327.60
HDL-C	48.75/48.67	13.75/13.82	8.25/8.00	111.00/108.50
LDL-C	118.52/117.64	35.77/34.68	3.75/3.20	338.20/335.20
HbA1C	7.94/7.92	1.45/1.49	4.20/4.50	16.20/15.90

In the table, “HDL-C” indicates high-density lipoprotein cholesterol and “LDL-C” indicates low-density lipoprotein cholesterol. In the column headers, “C/N” indicates T2D patient with cataract/T2D patient without cataract.

**Table 5:** Genotypic frequency distribution in cataract cases and controls.

**Chromosome**	**dbSNP ID**	**Physical position**	**Genotype**	**Cataract case n=109**	**Control n=649**	**OR**	**95% CI**	**p-value/Cp-value**
3	rs11129182	25146301	CC	56 (51.3)	487 (75.0)	1.00	Ref	** **
** **	** **	** **	CT	48 (44.0)	138 (21.3)	3.03	1.97–4.65	3.52×10^−7^/1.76×10^−6^
** **	** **	** **	TT	5 (4.6)	24 (3.7)	1.81	0.67–4.94	0.385
3	rs9876471	65848146	AA	4 (3.7)	0	** **	** **	4.48×10^−5^/2.24×10^−4^
** **	** **	** **	AG	12 (11.1)	103 (15.9)	0.69	0.37–1.31	0.321
** **	** **	** **	GG	92 (85.2)	546 (84.1)	1.00	Ref	** **
3	rs264668	65854285	CC	87 (79.8)	544 (83.8)	1.00	Ref	** **
** **	** **	** **	CT	18 (16.5)	105 (16.2)	1.07	0.62–1.86	0.916
** **	** **	** **	TT	4 (3.7)	0	** **	** **	2.79×10^−5^/1.40×10^−4^
3	rs17047573	68493809	GG	14 (12.8)	13 (2.0)	7.47	3.36–16.6	8.35×10^−8^/4.18×10^−7^
** **	** **	** **	GT	30 (27.5)	185 (28.5)	1.13	0.71–1.79	0.707
** **	** **	** **	TT	65 (59.6)	451 (69.4)	1.00	Ref	** **
3	rs17047586	68495564	AA	65 (59.6)	453 (69.9)	1.00	Ref	** **
** **	** **	** **	AG	30 (27.5)	182 (28.1)	1.15	0.72–1.83	6.43×10^−1^
** **	** **	** **	GG	14 (12.8)	13 (2.0)	7.51	3.38–16.7	7.65×10^−8^

In the table, “CI” indicates confidence interval. The χ2 test or Fisher's exact test was performed to obtain the p-value. The percentages of myopia and control with ht1 were compared with the percentages of myopia and control without ht1. Statistical significance was considered as p-value <0.05. The Cp-value is the p-value corrected by Bonferroni correction.

**Table 6:** Odds ratio and 95% CI for the association between haplotypes in 3p14.1- 3p14.2 and Myopia.

**Haplotype**			** **	** **	** **	** **
rs9876471	rs264668		**Cases**	**Control**	**OR (95% CI)**	**p-value/Cp-value**
G	C	Ht1	88.1%	91.9%	1.00 (Ref)	0.420/0.820
A	T	Ht2	9.6%	7.9%	1.27 (0.77–2.08)	** **
rs17047573	rs17047586	** **	** **	** **	** **	** **
T	A	Ht3	73.4%	83.7%	1.00 (Ref)	0.0002/0.0004
G	G	Ht4	26.6%	16.0%	1.89 (1.36–2.65)	** **

In the table, “CI” indicates confidence interval. The χ2 test or Fisher's exact test was performed to obtain the p-value. The percentages of myopia and control with ht1 were compared with the percentages of myopia and control without ht1. Statistical significance was considered as p-value <0.05. The Cp-value is the p-value corrected by Bonferroni correction.

## Discussion

The cut down of cataract level were LOCS 3 and 10 and the BCVA were 0.48 and 0.18 logMAR in this study. The patients who matched both the criteria of cataract level and visual acuity were included in the control and study groups. Because the patients with LOCS cataract scores of less than 3 or between LOCS 3–10 all had some form of cataract, the BCVA of the patients was also the including criteria. In addition, the progression of their cataracts might occur soon, the including criteria of cataracts and the control group was not a cut point. The patient with LOCS cataract scores of 3–10 were excluded from this study. In this study, we selected the patients with cataracts and poor BCVA as the study group and the patients with contrary criteria as the control group. The patient with senile cataracts could not be completely excluded; this was the limitation of our study. Nevertheless, the severity of the cataracts was the major criteria of the two groups. That is, the patients with the similar physical conditions and various severity of cataract were compared. A large amount of efforts has been devoted to finding common T2D genes, including genome-wide linkage, candidate-gene and genome-wide association studies [17]. Whole-genome linkage scans have identified chromosomal regions linked to T2D [18]. There are now at least 20 loci containing genes that increase the risk of T2D, including potassium channels (*KCNJ11* [potassium inwardly-rectifying channel, subfamily J, member 11] and *KCNQ1* [potassium voltage-gated channel, KQT-like subfamily, member 1]) [19], cell cycle regulators (*CDKAL1* [regulatory subunit associated protein 1-like 1], *CDKN2A-2B* [cyclin-dependent kinase inhibitor 2A-2B], and *CDC123-CAMK1D* [cell division cycle 123 homolog- calcium/calmodulin-dependent protein kinase ID]) [20], nuclear hormone receptor (*PPARG* [peroxisome proliferator-activated receptor gamma]) [21], melatonin receptor (*MTNR1B* [melatonin receptor 1B]) [22], transcription factors (*TCF7L2* [transcription factor 7-like 2], *TCF2* or *HNF1B* [hepatocyte nuclear factor 1 beta], *HHEX* [hematopoietically expressed homeobox], and *JAZF*1 [JAZF zinc finger 1]) [23], translational regulator (*IGF2BP2* [insulin-like growth factor 2 mRNA binding protein 2]) [24], zinc transporter (*SLC30A8* [solute carrier family 30 zinc transporter member 8]) [25], apoptosis components (*THADA* [thyroid adenoma associated]) [26], metalloprotease (*ADAMTS9* [ADAM metallopeptidase with thrombospondin type 1 motif, 9]) [27], calcium homeostasis component (*WFS1* [Wolfram syndrome 1 homolog]) [28], fat mass and obesity-associated gene (*FTO* [fat mass and obesity associated]) [29], pancreas organogenesis (*NOTCH2* [Notch homolog 2]) [30], and surface glycoprotein (*TSPAN8* [tetraspanin-8]) [31]. Variants in these genes are almost exclusively identified in population of European descent, and individually confer a modest risk (odds ratio=1.1–1.25) of developing T2D [31]. The ethnic differences in the genetic component are remarkable and the establishment of the genetic predisposition of our population is also important. The three SNPs with significant differences were found in cataract and T2D patients in this study, and were located at chromosome 3p14.1- 3p14.2. SNP rs11129182 with significant difference (p=3.52×10^−7^) in the patient with T2D and cataract simultaneously, and ‘*CT’* genotype was a risk marker (OR=3.03, 95% CI: 1.97–4.65) of the patients of T2D suffered cataract simultaneously. SNP rs11129182, located on chromosome 3p14.2, the gene and the genes locate in this region were with the function of regulate potassium voltage-gated channel, thyroid hormone receptors, and retinoic acid receptors. The high correlative rate of DM and cataracts in SNP rs11129182 was reasonable, but the exact effect of the SNP is worth elucidation. SNPs rs17047573 and rs17047586 (p value=8.35×10^−8^ and 7.65×10^−8^, individually; Table 3) expressed significant difference in the T2D patients with cataract. ‘GG’ genotype of rs17047573 and ‘GG’ genotype of rs17047586 were risk factors for T2D patients suffering from cataracts (7.47 and 7.51, individually; 95% confidence index (CI): 1.97–4.65, 3.36–16.6, and 3.38–16.7, individually). SNPs rs17047573 and rs17047586 are located at chromosome 3p14.1. The functions of the genes locates at chromosome 3p14.1 include voltage-dependent anion-selective channel protein, long myosin light chain kinase, adenylyl cyclase-associated protein, and retinoic acid receptor.

### T2D and potassium voltage-gated channel

In previous research by Unoki et al. [8], from 268,068 SNPs selected from the JSNP21 or HapMap database [32], potassium channels (*KCNJ11* and *KCNQ1*) were discovered to associate with the T2D in populations of both East Asian and European descent. *KCNQ1 *is located on chromosome 11p15.5 and is expressed in the pancreatic islets [33]. *KCNQ1* encodes the pore-forming subunit of the potassium channel. This evidence suggests that potassium channels might contribute to the pathogenesis of T2D [8,9].

### T2D and thyroid hormone receptor

Thyroid hormone analogs might be used to prevent or combat atherosclerosis, obesity and even T2D [34,35]. Current knowledge of thyroid hormone signaling suggests that thyroid hormone mimetics could worsen or improve different aspects of T2D. Thyroid hormone produces changes in hormone levels and gene profiles that resemble fasting states, in which insulin signaling should be reduced, and thyroid hormone stimulates liver gluconeogenesis, which increases blood glucose levels [36]. Conversely, thyroid hormone reduces body fat, reverses hepatic steatosis and increases mitochondrial activity [37,38], effects that could be protective against T2D.

### T2D and retinoic acid receptor

Neuropathy is the most common complication of DM; it occurs in 60% of patients and affects their quality of life [39,40]. Diabetic neuropathy is characterized by a progressive loss of nervous myelinic and amyelinic fibers. Retinoic acid had the significant effect of increasing neural growth factors in nerves when the other groups were compared to animals with DM [41]. Retinoic acid also regressed the ultrastructural changes induced by diabetes that showed increased neural regeneration [41]. RA can revert functional and ultrastructural changes and induce neural regeneration after the establishment of diabetic neuropathy. Retinoic acid is an essential molecule for cellular differentiation and an important morphogen in somatic development by binding to nuclear receptors and transcription complexes [41]. Other studies also indicated that retinoic acid might have renoprotective effects in the early stages of diabetic nephropathy through anti-inflammatory activity [42]. Moreover, one interesting study indicated that the serum levels of retinoic acids in diabetic patients correlated positively with the hemoglobin A1c (HbA1c) values. This evidence all supports the correlation between retinoic acid and DM [43].

### T2D and myosin light chain kinase

Diabetes-related cardiovascular disease remains the leading cause of death in patients with T2D [44]. Hypertension is common among diabetics and has the same pathogenetic mechanisms as insulin resistance, in which the activated renin–angiotensin system contributes to the increased high blood pressure and hyperglycemia. Hyperglycemia is one of the triggering factors for vascular dysfunction and clotting abnormalities, and therefore, for accelerated atherosclerosis in diabetes [44,45]. Furthermore, angiotensin-converting enzyme inhibitors might offer additional cardioprotection to diabetics beyond that provided by blood pressure reduction. The phosphorylation of myosin light chain in cardiac muscles by calmodulin-dependent kinase and has the modulatory role in the activation of myofibrillar adenosine triphosphatase (ATPase) and the process of force generation [44-47]. The myosin light chain is an important molecule in the diabetes-related cardiovascular disease [44-47].

### T2D and adenylyl cyclase-associated protein

Gia proteins are associated with functions in the aorta of the streptozotocin-induced diabetic rats [48]. A previous study by Hashim et al. [48] indicated that decreased levels and activity of Gi proteins and adenylyl cyclase signaling induced by hyperglycemia may be one of the important mechanisms contributing to the cardiovascular complications associated with diabetes [48,49].

### Cataract and the related proteins

The voltage-gated channel influences the intracellular iron concentrations that are closely related to denature of crystalline and influenced the cataract formation [50,51]. Thyroid hormones can confer a protective effect against oxidative damage, which is one of the main causes of damages to cells in the eye's lens and is related to the formation of cataracts [52-54]. Retinoic acid (RA)-mediated inhibition of deregulated calpains has effects on the development of cataracts [55]. RA inhibits Ca^2+^ elevation and subsequent overactivation of calpains, suggesting the potential feasibility of calpain-targeting therapies mediated by RA for cataracts [39-41]. MLC (myosin light chain) phosphorylation is noted to play an important role in maintaining lens function by regulating Rho kinase [55,56]. Adenylyl cyclase-associated protein was identified as a most efficient substrate of matrix metalloproteinases (MMP) [57]. Lens crystallins illustrates that intracellular structural proteins are MMP substrates. Adenylyl cyclase-associated protein can induce the rapid turnover rate of MMP-9 [57]. Lens opacity may be related to adenylyl cyclase-associated protein [57]. These protein all are related to the cataractogenesis and the pathogenesis of T2D. In the haplotype study, Ht4 was significantly higher in the cases group and with an odd ratio: 1.89 and 95% CI:1.36–2.65. This meant that Ht4 was a risk factor for susceptibility to cataracts and DM. This evidence makes us realize the correlation between rs11129182, rs17047573, and rs17047586 with T2D and cataract and helps us in predicting and designing new diagnoses and treatment methods for T2D patients with cataracts.
